# Highly Pathogenic PRRSV-Infected Alveolar Macrophages Impair the Function of Pulmonary Microvascular Endothelial Cells

**DOI:** 10.3390/v14030452

**Published:** 2022-02-22

**Authors:** Weifeng Sun, Weixin Wu, Nan Jiang, Xinna Ge, Yongning Zhang, Jun Han, Xin Guo, Lei Zhou, Hanchun Yang

**Affiliations:** Key Laboratory of Animal Epidemiology of Ministry of Agriculture and Rural Affairs, College of Veterinary Medicine, China Agricultural University, Beijing 100193, China; sunwf@cau.edu.cn (W.S.); werson@cau.edu.cn (W.W.); jiangnan314@126.com (N.J.); gexn@cau.edu.cn (X.G.); zhangyongning@cau.edu.cn (Y.Z.); hanx0158@cau.edu.cn (J.H.); yanghanchun1@cau.edu.cn (H.Y.)

**Keywords:** porcine reproductive and respiratory syndrome virus (PRRSV), pulmonary microvascular endothelial cells (PMVECs), transcriptome analysis, transwell co-cultures, cytokines, tight junctions (TJs)

## Abstract

The porcine reproductive and respiratory syndrome virus (PRRSV), especially the highly pathogenic strains, can cause serious acute lung injury (ALI), characterized by extensive hemorrhage, inflammatory cells and serous fluid infiltration in the lung vascular system. Meanwhile, the pulmonary microvascular endothelial cells (PMVECs) are essential for forming the air–blood barrier and keeping the water–salt balance to prevent leakage of circulating nutrients, solutes, and fluid into the underlying tissues. As well, they tightly regulate the influx of immune cells. To determine the possible relationship between the PMVECs’ function changes and lung vascular permeability during PRRSV infection, the PMVECs were co-cultured with HP-PRRSV-inoculated primary pulmonary alveolar macrophages (PAMs) in transwell model, and then the RNA sequencing (RNA-seq) and comprehensive bioinformatics analysis were carried out to characterize the dynamic transcriptome landscapes of PMVECs. In total, 16,489 annotated genes were identified, with 275 upregulated and 270 downregulated differentially expressed genes (DEGs) were characterized at both 18 and 24 h post PRRSV inoculation. The GO terms and KEGG pathways analysis indicated that the immune response, metabolic pathways, cell death, cytokine–cytokine receptor interaction, viral responses, and apoptotic process are significantly regulated upon co-culture with PRRSV-infected PAMs. Moreover, according to the TERR and dextran flux assay results, dysregulation of TJ proteins, including CLDN1, CLDN4, CLDN8, and OCLN, is further confirmed to correlate with the increased permeability of PMVECs. These transcriptome profiles and DEGs will provide valuable clues for further exploring the roles of PMVECs in PRRSV-induced ALI in the future.

## 1. Introduction

Porcine reproductive and respiratory syndrome virus (PRRSV) is classified into the genus *Betaarterivirus*, family *Atreriviridae*, and order *Nidovirales* [1]. It is the pathogen of the porcine reproductive and respiratory syndrome (PRRS), which is characterized by severe reproductive failure in sows, respiratory disorder, reduction in growth rate, and increased mortality in the outbreak herd. Since the first report in the late 1980s [2,3,4,5], PRRS has become one of the most economically important swine diseases that seriously hinders the development of the pork industry worldwide. Especially, some “atypical” highly pathogenic strains, causing extended severity and ranges of clinical signs, periodically emerge in Asia, East Europe, and America. Notably, the highly pathogenic PRRS (HP-PRRS), known as “porcine high fever diseases”, associated with high body temperature (as high as 42 °C), high mobility, high mortality, and devastating economic loss, emerged in China in 2006 and spread in several Southeast Asian countries in the following years [6,7,8,9]. Similarly, another highly pathogenic variant, Lineage 1C strain, typed as 1-4-4 pattern of restriction fragment length polymorphism (RFLP) for its open reading frame 5 (ORF5) gene, has been recently reported in many midwestern states of the United States. During the outbreak, the herds show increased farrow-to-finish mortality, abortions, mummies, and slower growth in finishing pigs [10]. The epidemic of these variant strains has greatly reformed the understanding of pathogenicity and the economic impact of PRRSV.

PRRSV can cause interstitial pneumonia in all ages of pigs, with varied severity and distribution as the difference of viral virulence, host susceptibility, and host immune status. Among the PRRSV-induced microscopic lesions, it is consistently observed that the alveolar septa are expanded by macrophages, lymphocytes, and plasma cells; as well, the alveoli are filled with necrotic macrophages, cell debris, and fluid. For HP-PRRSV infection, acute lung injury (ALI) is widely observed, which is characterized by aberrant immune responses, involving extensive hemorrhage and infiltration of inflammatory cells and serous fluid in the lung vascular system [11]. These severe lesions might contribute to the increased mortality of HP-PRRS. However, the underlying mechanisms of ALI caused by HP-PRRSV remain unclear, such as how the virus infection induces the circulating inflammatory cells and erythrocytes, as well as fluid flux into the sub-endothelial space.

Together with basement membrane and perivascular cells, the endothelial cells in the medial surfaces of blood vessels constitute an intact vascular barrier [12]. The primary function of the vascular barrier is to prevent leakage of circulating nutrients, solutes, and fluid into the underlying tissues. As well, it can tightly regulate the influx of immune cells [13]. Intercellular junctions among the adjacent endothelial cells provide blood and lymph vessel integrity, and they are essential for the formation of a vascular system, which controls the paracellular movement of the substances above and through the endothelium [12]. Altered endothelial junctions can lead to barrier dysfunction and have been implicated in several kinds of diseases, such as severe infections, cancer, and aggressive inflammatory responses [14]. One devastating manifestation of the disassembly of endothelial junctions is observed in ALI. The excessive immune response triggers the disruption of the lung endothelial barrier, and fluid and protein leak out of the lung capillaries and flux into the alveolar space, causing lung edema. Then, the fluid impairs gas exchange across the air–blood barrier and compromises respiratory function [15,16]. Various transmembrane adhesion proteins are located at the adherents and tight junctions, to connect adjacent endothelial cells and to sustain the endothelial barrier integrity through homophilic interactions [17]. On the intracellular side, adhesion proteins cytoplasmic tails interact with actin cytoskeleton and adherents or tight junction-associated proteins, including β-catenin, α-catenin, zona occludens 1 (ZO-1) and 2 (ZO-2), and others to stabilize the junctions [12,17,18]. Considering the pivotal roles of endothelial junctions on barrier integrity, we wonder whether these proteins in endothelial cells are regulated when PRRSV induces the ALI.

In addition to maintaining endothelial barrier integrity, the endothelial cells are also important for inflammatory responses [19,20]. During lipopolysaccharide (LPS) stimulation, virus infection, inflammation, and tissue injury, endothelial cells can secrete a high level of chemokines and cytokines, such as tumor necrosis factor α (TNF-α) and interleukin-1 (IL-1). Meanwhile, on the surface of vascular endothelial cells, the leukocyte adhesion molecules, particularly the vascular cell adhesion molecule-1 (VCAM-1) and intercellular adhesion molecule 1 (ICAM-1), are upregulated, aggravating inflammation and promoting monocyte extravasation from vessels into perivascular tissues [21,22,23,24]. As the primary target cells of PRRSV, PAMs can secrete abundant cytokines and chemokines in the course of infection, such as IL-1, TNF-α, and RANTES [25,26]. Thus, the PRRSV-Infected PAMs might interact with PMVECs to impair the air–blood barrier; however, the role of PMVECs in PRRSV-induced ALI is less known.

In the present study, the role of PMVECs on the PRRSV-induced ALI is investigated by establishing PMVECs and PAMs transwell co-culture system and using RNA-seq analysis. The results initially provided an overall transcriptome landscape of PMVECs that interact with HP-PRRSV strain JXwn06-infected PAMs, and the deeper analyses further demonstrate that the interaction can dysregulate the tight junction (TJ) proteins and facilitate chemokines as well as leukocyte adhesion molecule production in PMVECs. These results provide important insights into the mechanisms of lung vascular permeability changes during PRRSV infection.

## 2. Materials and Methods

### 2.1. Ethical Statements

The animal experiments in this study were carried out according to the Chinese Regulations of Laboratory Animals: The Guidelines for the Care of Laboratory Animals (Ministry of Science and Technology of the People’s Republic of China, Beijing, China) and Laboratory Animal Requirements of Environment and Housing Facilities (National Laboratory Animal Standardization Technical Committee). The protocol for primary PAMs preparation was approved by the Laboratory Animal Ethical Committee of CAU, with approval no. AW81801202-2-1.

### 2.2. Cells and Virus

Primary PAMs were prepared from 4-week-old specific-pathogen-free (SPF) landrace pigs, as previously described [4,27,28]. The pigs were purchased from the Beijing Center for SPF Swine Breeding and Management that is free from PRRSV, African swine fever virus (ASFV), porcine circovirus type 2 (PCV2), classical swine fever virus (CSFV), pseudorabies virus, swine influenza virus, and *Mycoplasma hyopneumoniae* infection. Briefly, the lavage fluid was collected from the lungs of euthanized pigs and washed about ten times with PBS supplemented with 2% fetal bovine serum (FBS) (Thermo Fisher, Waltham, MA, USA). The cell pellets were resuspended and mixed with prechilled GIBCO RPMI-1640 medium (Thermo Fisher, Waltham, MA, USA) containing 40% FBS. The number of the prepared PAMs reached 10^8^–10^9^ /mL with >95% viability. Aliquots of PAMs were frozen and stored in liquid nitrogen before use. The viability of PAMs was determined to be 85–90% by trypan blue dye exclusion. PAMs were maintained in GIBCO PRMI-1640 medium, with 10% FBS, 100 mg/mL kanamycin, 50 U/mL penicillin, 50 mg/mL streptomycin, 25 mg/mL polymyxin B, and 1 mg/mL fungizone at 37 °C under a humid 5% CO_2_ atmosphere. The immortalized endothelial cell line PMVEC (YaJi Biological, YS1234C, Shanghai, China) was cultured in RPMI-1640 medium supplemented with 5% FBS at 37 °C under a humid 5% CO_2_ atmosphere. The HP-PRRSV strain JXwn06 (GenBank accession number EF641008) at the 8th passage was used in this study [9].

### 2.3. Transwell Co-Cultures System

A total of 2.5 × 10^5^ PMVECs was seeded into transwell inserts with 24 mm diameter, 0.4 µm pore size (Corning Inc., Corning, NY, USA), and was allowed to grow in RPMI-1640 medium supplemented with 5% FBS to confluence. Primary PAMs were plated into the basolateral chamber about 12 h before PMVECs became confluent. Upon the PMVEC monolayer growing to confluence, the PAMs were inoculated with HP-PRRSV strain JXwn06 at a multiplicity of infection (MOI) of 5 or treated with RPMI-1640 medium as mock-infection. After 1 h incubation, the virus inoculum was carefully removed, and the cells were washed with PBS and further maintained in RPMI-1640 medium containing 2% FBS. At 18 and 24 h post infection (hpi), the supernatant was discarded, and the PMVECs in both groups were immediately harvested for RNA extraction by using TRIzol (Life Technologies, Carlsbad, CA, USA) and RNA-seq for transcriptomic analysis.

### 2.4. Transcriptome mRNA Library Construction and Sequencing

Total RNA was extracted from PMVECs in different groups using TRIzol reagent, following the manufacturer’s procedure. RNA library construction and sequencing were performed by LC Bio (Zhejiang, China). Briefly, the total RNA quantity and purity were analyzed by Bioanalyzer 2100 and RNA 1000 Nano LabChip Kit (Agilent, Santa Clara, CA, USA) with RIN number > 7.0. Poly(A) RNA is purified from total RNA (5 µg) using poly-T oligo-attached magnetic beads with two rounds of purification. After purification, the mRNA is fragmented into small pieces using divalent cations under elevated temperatures. Then, the cleaved RNA fragments were reverse-transcribed to create the final cDNA library following the protocol for the mRNA-Seq sample preparation kit (Illumina, San Diego, CA, USA). The average size of insert for the paired-end libraries was 300 bp (±50 bp). Then, the paired-end sequencing was performed on an Illumina Hiseq4000 (LC Sciences, Houston, TX, USA) following the vendor’s recommended protocol. The raw sequencing data (raw reads) were preserved in FASTQ format. Clean reads were obtained by removing the adaptors, reads of the unknown base with more than 10%, and those with low quality from the raw reads. The acquired reads were then aligned to the *Sus scrofa* genome assembly (*Sus scrofa* 10.2) using TopHat2 [29]. The mapped reads of each sample were assembled by using StringTie. Then, all transcriptomes from samples were merged to reconstruct a comprehensive transcriptome using Perl scripts. After the final transcriptome was generated, StringTie and edgeR were used to estimate the expression levels of all transcripts by calculating fragments per kilobase of exon model per million mapped (FPKM) reads. The differentially expressed mRNAs and genes were selected with log2 (fold change) > 1 or log2 (fold change) < −1 and with statistical significance (*p* value < 0.05) by the R package.

### 2.5. Venn, GO and KEGG Pathway Enrichment Analysis

Venn analysis, GO enrichment, and KEGG enrichment analysis were performed as described by LC Bio (https://www.lc-bio.cn/, accessed on 11 January 2022).

### 2.6. Quantitative Reverse Transcription PCR (RT-qPCR)

PMVECs were collected from the co-culture system at 18 and 24 hpi, and the total RNAs were extracted by using TRIzol following the manufacturer’s instructions. Then, 1 µg of RNAs was used for further reverse transcription utilizing the FastKing RT Kit (with gDNase) (TIANGEN, KR116, Beijing, China). Quantitative PCR (qPCR) was performed by using Bio-Rad CFX96 Touch Real-Time PCR cycler (Bio-Rad, Hercules, CA, USA) with primers listed in Table 1 and SYBR green detection, with the thermal protocol: 50 °C for 5 min; 95 °C for 2 min; followed by 40 cycles of 95 °C for 10 s and 60 °C for 50 s. Data collection was performed at the step of 60 °C annealing/elongation. Relative quantification of target genes was performed using the 2^−ΔΔCt^ method with β-actin as a housekeeping gene.

### 2.7. Western Blot

Extraction of total proteins from treated PMVECs was performed with RIPA lysis buffer (Beyotime, P0013B, Shanghai, China) supplemented with 1 mM PMSF (Beyotime, ST506, Shanghai, China) on ice. Protein concentrations were measured with an enhanced BCA protein assay kit (Beyotime, P0010S, Shanghai, China). Then, 20 ug proteins per sample was mixed with 5 × loading buffer and boiled at 70 °C for 15 min, and they were separated by SDS-PAGE. After transferring the proteins onto a polyvinylidene difluoride membranes (PVDF, Millipore, IPVH07850, Darmstadt, Germany), the membrane was blocked in phosphate-buffered saline (PBS) with 5% skimmed milk at room temperature for 2 h, followed by incubation at 4 °C overnight with primary antibodies and then horseradish peroxidase (HRP)-conjugated anti-mouse or anti-rabbit secondary antibodies at room temperature for 1 h while shaking. The protein bands were detected by the ECL Western blotting system (Thermo Fisher, Waltham, MA, USA). The following primary antibodies were used: anti-CLDN1 (1:2000, Proteintech, 13050-1-AP, Rosemont, IL, USA), anti-CLDN4 (1:1000, Abcam, ab53156, Cambridge, UK), anti-CLDN8 (1:1000, Novus Biologicals, NBP1-59157, Littleton, CO, USA), anti-OCLN (1:1000, CUSABIO, CSB-PA016263LA01HU, Hubei, China), and anti-ACTB (1:5000, CUSABIO, CSB-MA000091M0m, Hubei, China).

### 2.8. Trans-Endothelial Electrical Resistance and In Vitro Vascular Permeability Assay

The integrity of the PMVECs monolayer was first evaluated by measuring trans-endothelial electrical resistance (TEER). In transwell cultures, TEER was measured by using an Epithelial Volt Ohm Meter (EVOM) with “chopstick” electrodes (Beijing Kingtech Technology, RE1600, Beijing, China) as previously described [30]. Inserts with medium alone were used for blank resistance measurements. TEER values (Ω·cm^2^) of PMVEC monolayer at different time points were measured and calculated according to the following formula:

$$\mathrm{TEER}=\left(\Omega_{\mathrm{endothelial}\;\mathrm{cells}}-\Omega_{\mathrm{medium}\;\mathrm{alone}}\right)\times \mathrm{cell}\;\mathrm{culture}\;\mathrm{surface}\;\mathrm{area}$$

To evaluate the integrity of the PMVEC barrier post virus infection in vitro, the TEER and vascular permeability assay were both performed. First, pulmonary microvascular endothelial cells were grown until TEER values ranged between 15 and 18 (Ω·cm^2^), indicating 100% cell confluency. Then, the primary PAMs plated in the basolateral chamber were inoculated with JXwn06 or mock at an MOI of 5. At sequential 12 h time points post inoculation, the TEER values, expressed in Ohms (Ω), were tested by using EVOM. Endothelial permeability was expressed as relative TEER, which represents a ratio of resistance values (Ω) as follows:

$$\mathrm{Relative}\;\mathrm{TEER}=\frac{\Omega_{\mathrm{expreimental}\;\mathrm{condition}}-\Omega_{\mathrm{medium}\;\mathrm{alone}}}{\Omega_{\mathrm{non}-\mathrm{treated}\;\mathrm{endothelial}\;\mathrm{cells}}-\Omega_{\mathrm{medium}\;\mathrm{alone}}}\times 100\%$$

At the same time, transwell inserts were transferred to another 12-well plate supplemented with 1.5 mL Hank’s balanced salt solution (HBSS), and then 0.5 mL 40 kDa dextran conjugated to FITC (Sigma Aldrich, St. Louis, MO, USA) was added to the apical chamber of the transwell inserts at a final concentration of 1 mg/mL and incubated for 2 h at 37 °C. Then, the transwell inserts were removed, and 100 µL supernatant from each well was collected from triplicate wells and transferred to a 96-well flat-bottom plate. Fluorescence was measured on a plate reader, and the concentration of dextran–FITC that passed from the apical to the basolateral chamber was determined by using the standard curve (3.125–50 µg/mL). The PMVECs monolayers in the mock-infected group were used as baseline control.

### 2.9. Statistical Analysis

The data from RT-qPCR, RNA-seq, TEER as well as in vitro vascular permeability assay were shown as means ± standard deviations (SD). The GraphPad Prism software (version 5.0) was used to determine the significance of the variability among different groups by a two-way ANOVA test of variance. A *p* value < 0.05 was considered to be statistically significant.

## 3. Results

### 3.1. Establishment of PMVECs and PAMs Transwell Co-Cultures In Vitro

**Figure 1:**
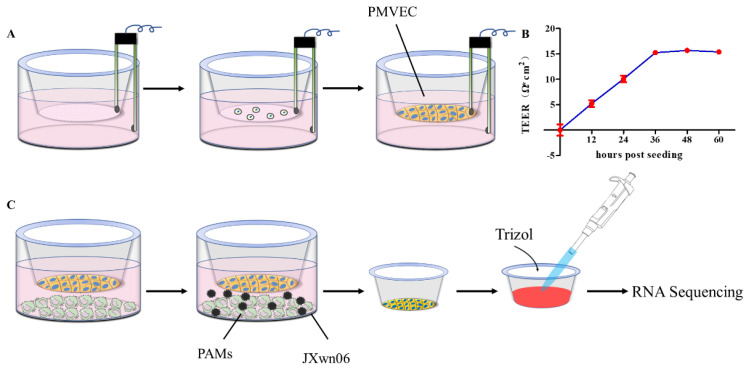
Schematic for PMVECs and primary PAMs transwell co-cultures and the preparation of samples for RNA-seq. (**A**) PMVECs were plated onto transwell semi-permeable membranes (0.4 μm pore size), and inserts with medium alone were used for blank resistance measurements. A TEER assay was used to evaluate the integrity of the PMVECs monolayer at indicated time points over 60 h. (**B**) Relative TEER values from three independent experiments performed in triplicate are plotted. The data are shown as means ± SD (standard deviation). (**C**) Upon the PMVEC monolayer grown to confluence, primary PAMs were infected with HP-PRRSV JXwn06 at an MOI of 5 or treated with mock-infected. Total RNA of PMVECs was extracted by using TRIzol reagent at different time points post viral infection for RNA-seq analysis.

To investigate the functional changes of PMVECs during PRRSV infection, a transwell co-culture system with PAMs was set to mimic the endothelial barrier in vitro. PMVECs were seeded on the apical chamber of transwell inserts, and the integrity of the monolayer was evaluated by measuring TEER at different time points after cells were plated (Figure 1A), which was regarded as the standard parameter to quantify the tightness of the endothelial barrier [30,31]. As shown in Figure 1B, the TEER of PMVECs monolayer rose continuously during 12–36 h post-seeding, followed by a plateau lasting for an additional 24 h with the TEER values around 15 to 18 Ω·cm^2^, indicating 100% cell confluency. This was also verified by the FITC–Dextran transwell assay in vitro (data not shown). These data suggest that the PMVEC monolayer cultured in vitro can form a tight endothelial barrier after 36 h post-seeding. Upon the PMVEC monolayer grown to confluence, they were then co-cultured with primary PAMs (Figure 1C)

### 3.2. Differential Transcription Analysis of Genes in PMVECs in Response to the Interaction with HP-PRRSV-Infected PAMs

To evaluate the effects of HP-PRRSV-infected PAMs on the endothelial barrier, the macrophages were inoculated with HP-PRRSV strain JXwn06 at an MOI of 5 to analyze how they modulate endothelial barrier integrity (Figure 1C). Total RNA of PMVECs was extracted by using TRIzol Reagent at 18 and 24 hpi respectively, followed by RNA-seq to detect the mRNA transcription profiles in two groups (JXwn06-infected group vs. mock-infected group) (Figure 1C right).

**Figure 2:**
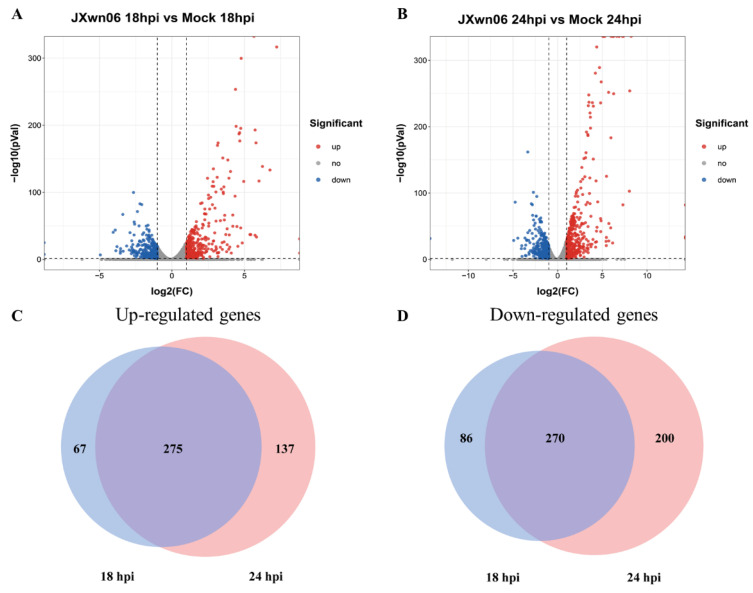
Differentially expressed genes (DEGs) in PMVECs co-cultured with HP-PRRSV strain JXwn06-infected PAMs. In RNA-seq analysis, three independent experiments were repeated in each group. (**A**,**B**) Volcano map of a distinguishable mRNA expression profiling in PMVECs after JXwn06 inoculation at 18 and 24 hpi, respectively. The differentially expressed genes were selected with log2 (fold change) > 1 or log2 (fold change) < −1 and with statistical significance (*p* value < 0.05) by R package. (**C**,**D**) Veen analysis was performed to identified genes co-regulated by JXwn06 at 18 hpi and 24 hpi, including 275 co-upregulated (**C**) and 270 co-downregulated (**D**) genes. Data were extracted from RNA-seq results.

The transcriptome analysis results showed that 16,489 genes were identified in total. In comparison with the mock-infected group, there were 340 upregulated genes and 354 downregulated genes in the inoculation group at 18 hpi (Figure 2A), which increased to 409 and 469 genes at 24 hpi (Figure 2B), according to the statistical criteria of log2 (fold change) > 1 or log2 (fold change) < −1 and statistical significance (*p* value < 0.05). Among them, there were 275 upregulated genes (Figure 2C) as well as 270 downregulated genes (Figure 2D) conserved at these two time points.

Among these conserved genes at the two time points, the most enriched upregulated ones are mainly involved in positive regulation of cell migration, including vascular cell adhesion molecule-1 (VCAM-1), Claudin 4 (CLDN4), C-C motif chemokine ligand 20 (CCL20), CCL22, C-X3-C motif chemokine ligand 1 (CX3CL1). Besides, several genes involved in innate immune response were also upregulated in PMVECs, including IL-1α, colony-stimulating factor 3 (CSF3), chemokine CCL20, and STAT1, which have been previously reported to be involved in PRRSV-infected PAMs as well [26,32,33]. Meanwhile, several genes such as hyaluronidase 3, gap junction protein beta 1, aquaporin 3, transferrin, and CLDN8, involved in cell junctions, ion delivery, water, and glycerol permeation, were found to be significantly downregulated at about 74–94%, compared with that in the mock-infected group. Few downregulated genes were related to immune response, which is different from the transcriptome data of PAMs, showing great immune suppression after PRRSV infection [28].

### 3.3. Analysis of Gene Ontology (GO) Terms and Kyoto Encyclopedia of Genes and Genomes (KEGG) Pathways of DEGs

**Figure 3:**
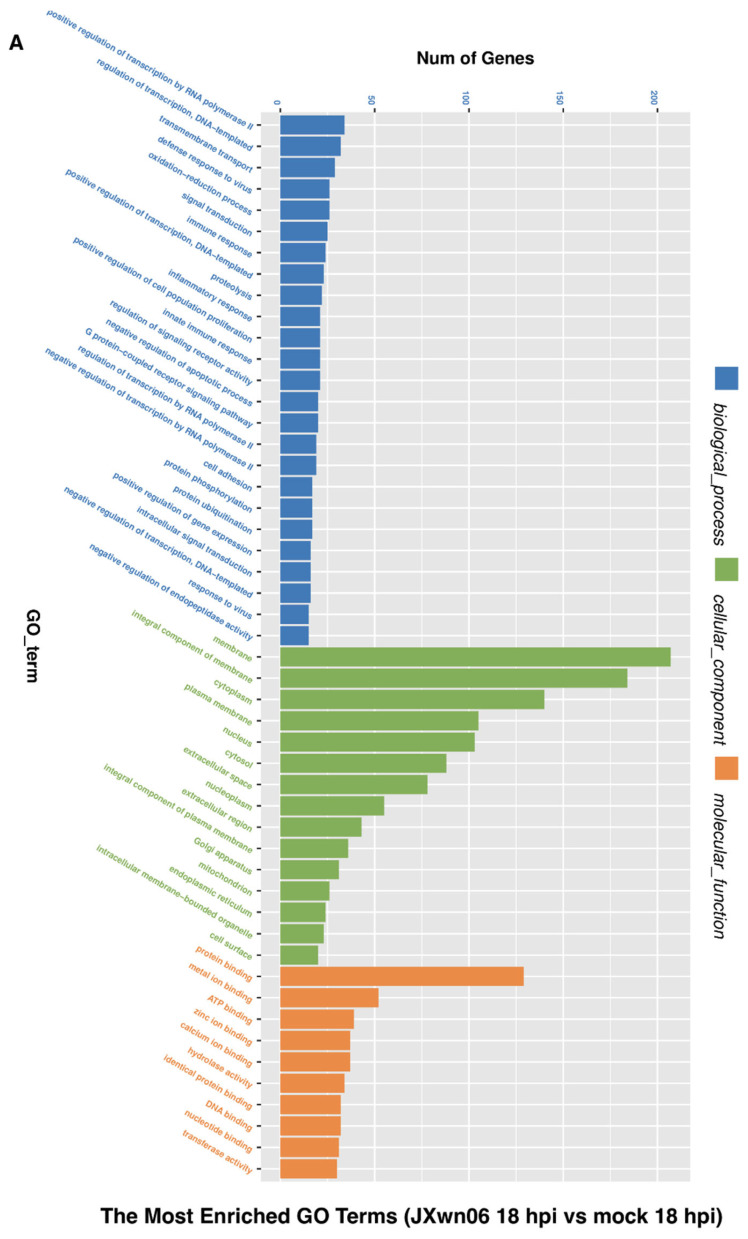

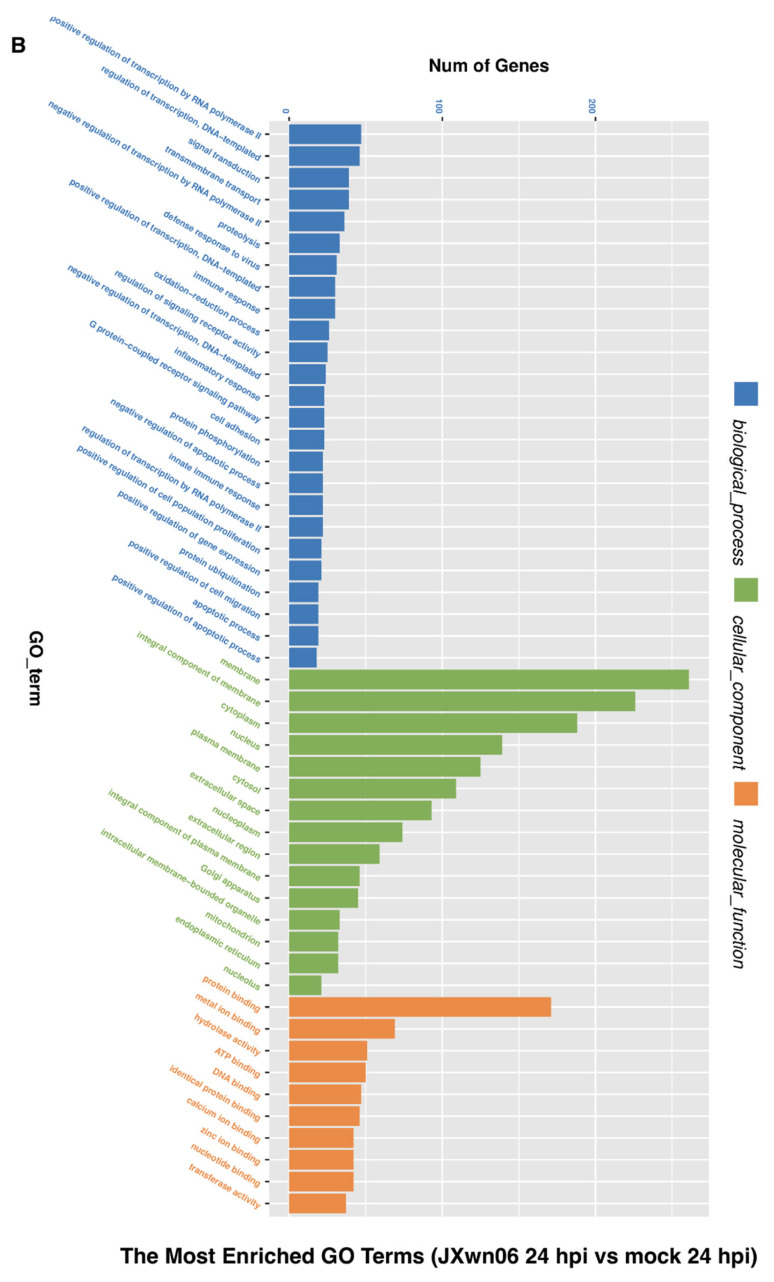

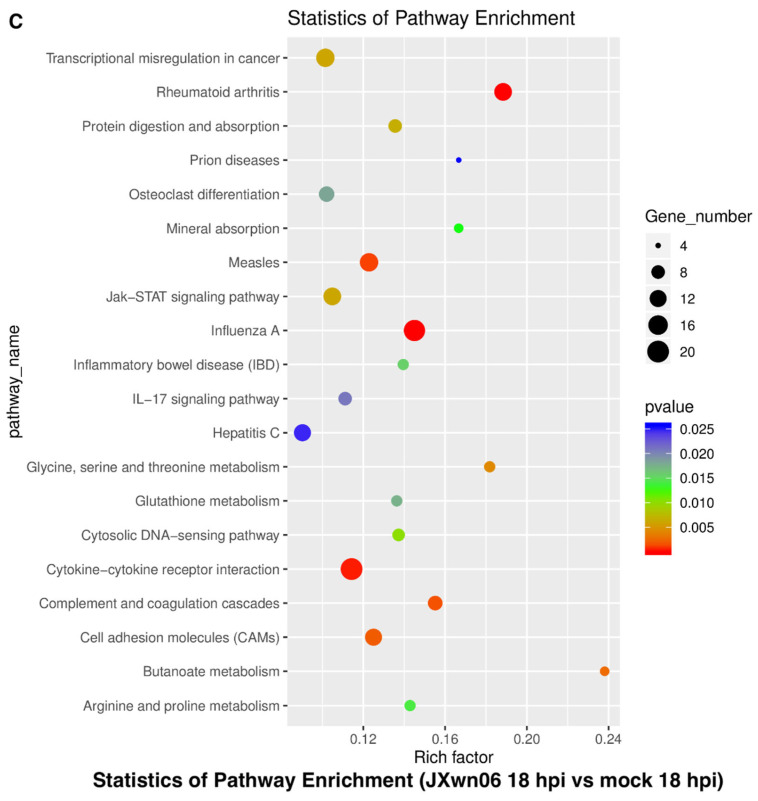

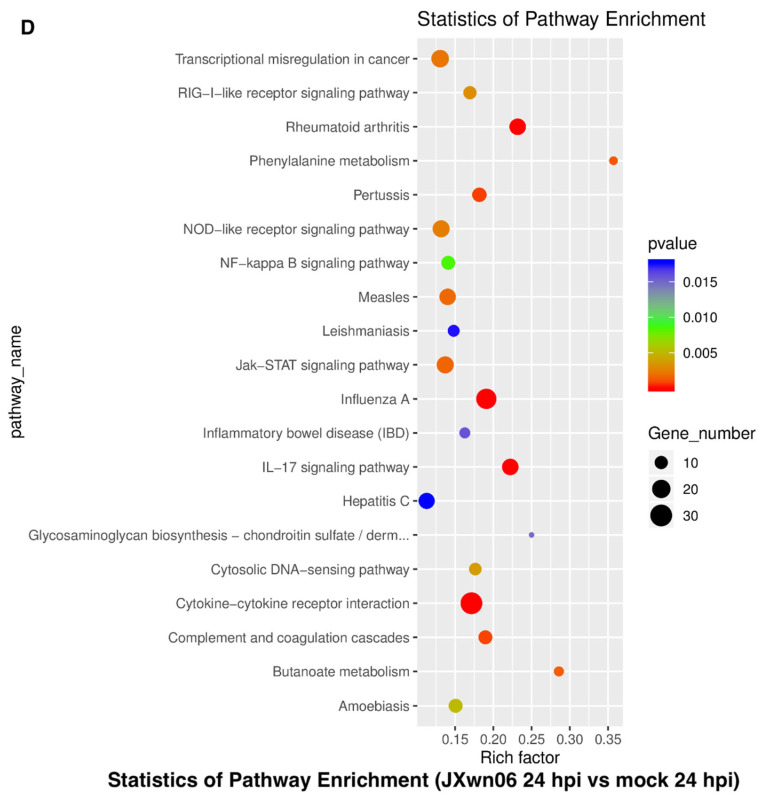
Gene Ontology (GO) terms and KEGG pathway enrichment of the DEGs. (**A**,**B**) The most significant enriched GO terms (top 50) among the DEGs in the JXwn06 inoculation group at 18 hpi vs. mock 18 hpi (**A**) and JXwn06 inoculation group at 24 hpi vs. mock 24 hpi (**B**). (**C**,**D**) The most significant enriched KEGG pathways among the DEGs in the JXwn06 inoculation group at 18 hpi vs. mock 18 hpi (**C**) and JXwn06 inoculation group at 24 hpi vs. mock 24 hpi (**D**).

As the gene dysregulation reflects the molecular phenotype of PMVECs affected by the HP-PRRSV-infected PAMs, the GO terms and KEGG pathways analysis were also carried out to further determine the functions of DEGs in PMVECs. The basic functions of the top altered genes were classified into three terms, including biological process, cellular component, and molecular function (Figure 3A,B). The biological processes of enriched GO terms include regulation of transcription, signal transduction, defense response to the virus, immune response, and cell adhesion as well as positive or negative regulation of apoptosis. For the cellular component, the membrane, integral component of membrane, and cytoplasm were identified as the top three items for the infection group. Most molecular functions identified were classified into several “binding” activities. The major processes of enriched GO terms were conserved between 18 and 24 hpi.

The altered pathways associated with dysregulated genes in the HP-PRRSV-infected group were further represented (Figure 3C,D). Cytokine–cytokine receptor interactions, NF-κB, Jak-STAT, and PI3K-Akt signaling pathway, as well as monocyte adhesion and metabolism pathways, were dysregulated upon interaction with HP-PRRSV-infected PAMs. Collectively, the data suggest that the interaction majorly regulated the cytokine activation, monocyte adhesion, NF-κB signaling pathway, and cell adhesion pathways in PMVECs, which might be the response to the cytokines secreted by PAMs.

### 3.4. Cell Adhesion Molecules and Pro-Inflammatory Cytokines Are Induced in PMVECs upon the Interaction with HP-PRRSV-Infected PAMs

**Figure 4:**
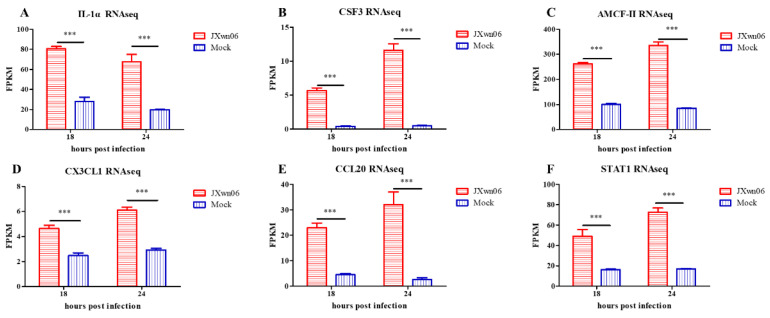
Analysis of several DEGs associated with cytokine–cytokine receptor interactions and chemokine signaling pathway. RNA-seq data in fragments per kilobase million (FPKM) for (**A**) IL-1α, (**B**) CSF3, (**C**) AMCF-II, (**D**) CX3CL1, (**E**) CCL20, and (**F**) STAT1 for comparison are presented. The data are shown as means ± SD (standard deviation), n = 3 independent experiments. Asterisks indicate statistical significance (***, *p* < 0.001).

To evaluate the roles of PMVECs in HP-PRRSV-induced inflammatory responses, a comprehensive analysis specifically on monocyte adhesion pathways and pro-inflammatory cytokine induction was further performed. Compared with the mock-infected group, the transcription of IL-1α, CSF3, AMCF-II, CX3CL1, CCL20, and STAT1 genes (Figure 4) in PMVECs from the HP-PRRSV infection group were strongly upregulated, suggesting that inflammatory responses were well induced upon the interaction with HP-PRRSV-infected PAMs. Conversely, intercellular adhesion molecule-1 (ICAM-1) and vascular cell adhesion molecule-1 (VCAM-1) were also upregulated in the HP-PRRSV infection group, indicating that it may facilitate monocyte adhesion and rolling along the vascular wall by promoting adhesion molecule expression on the surface of PMVECs.

Previous studies have reported that PRRSV infection induces pro-inflammatory cytokine production, such as IL-6, IL-8, and TNF-α, by activating the NF-κB signaling pathway [33,34]. In addition, IL-1α was always higher in the lungs of PRRSV-inoculated animals, which was correlated with the severity of pulmonary lesions [26]. Although PMVECs are unsusceptible to PRRSV, our results convincingly demonstrate that they are an important type of cell in inflammatory responses during PRRSV infection, which exacerbate inflammatory injury by secreting inflammatory cytokines into the lung microenvironment [19,20].

In a previous inoculation study, severe histopathological lesions were usually observed in the lungs of HP-PRRSV-infected pigs, including a large number of inflammatory cell infiltration [11]. For the PMVECs, except for releasing pro-inflammatory cytokines and chemokines, they can also display leukocyte adhesion molecules on the surface of endothelial cells to initiate monocyte adhesion and rolling along the vascular wall. Taken together, our results reveal that HP-PRRSV-infected PAMs can interact with PMVECs and activate several genes transcriptions mainly manifested as excessive inflammatory responses and high levels of adhesion molecules, which might contribute to the monocyte chemotaxis in PRRSV-induced lung lesions.

### 3.5. HP-PRRSV Triggers Endothelial Barrier Dysfunction In Vitro

It is known that the movement of leukocytes through the endothelium is tightly regulated by the dynamic opening and closure of junctions between the adjacent endothelial cells [12]. Given that HP-PRRSV infection results in a large number of inflammatory infiltration and extensive hemorrhage [11], it is speculated that endothelial barrier integrity might be destroyed to promote leakage of circulating immune cells and erythrocytes into the tissues. Next, the roles of HP-PRRSV in the PMVEC barrier dysfunction were evaluated in the co-culture system.

**Figure 5:**
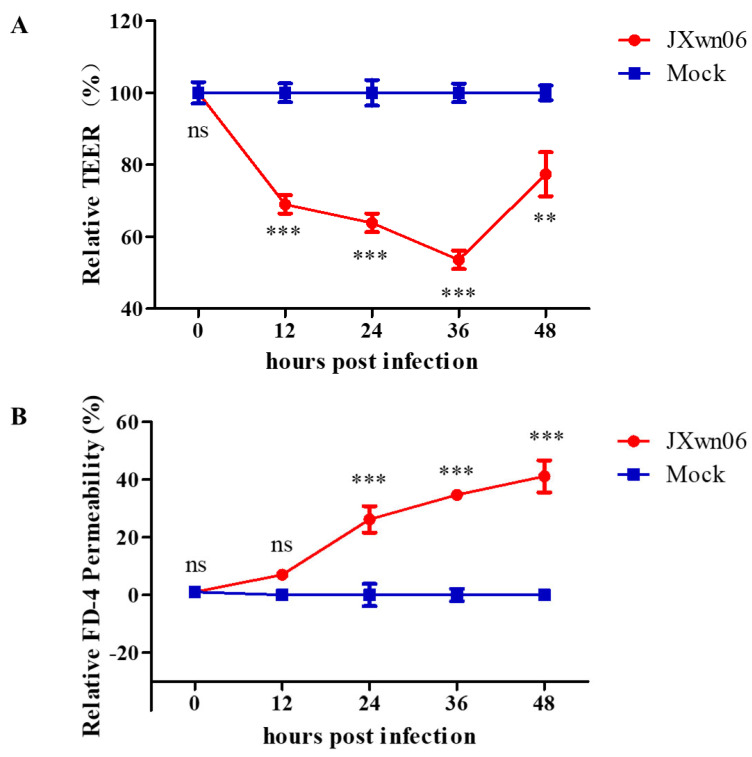
JXwn06 triggers endothelial barrier dysfunction in vitro. PMVECs were grown on transwell semi-permeable membranes (0.4 μm pore size) and allowed to grow to confluence. Then, the primary PAMs plated in the basolateral chamber were inoculated with JXwn06 at an MOI of 5, and mock-infected groups were set as a negative control. The permeability of the PMVEC monolayer was determined by TEER (**A**) and FITC–Dextran transwell assay (**B**). The data are shown as means ± SD (standard deviation), n = 3 independent experiments performed in triplicate. Asterisks indicate statistical significance (ns, *p* > 0.05; **, *p* < 0.01; ***, *p* < 0.001).

Here, a TEER assay was used to evaluate the ability of HP-PRRSV-infected PAMs to trigger the endothelial barrier dysfunction in PMVECs. The results show that the infection of HP-PRRSV in the co-culture system triggers the hyperpermeability of PMVECs in vitro, which was manifested by the dramatic decline in TEER values, as early as 12 h post HP-PRRSV infection (Figure 5A). The TEER values were further confirmed in a solute flux assay using macromolecules at 40 kDa dextran conjugated to FITC as a tracer in PMVEC monolayers (Figure 5B). Taken together, the integrity of the pulmonary microvascular endothelial barrier is compromised in vitro upon interacting with HP-PRRSV-infected PAMs.

### 3.6. HP-PRRSV Infection Dysregulates PMVEC Tight Junction Proteins In Vitro

**Figure 6:**
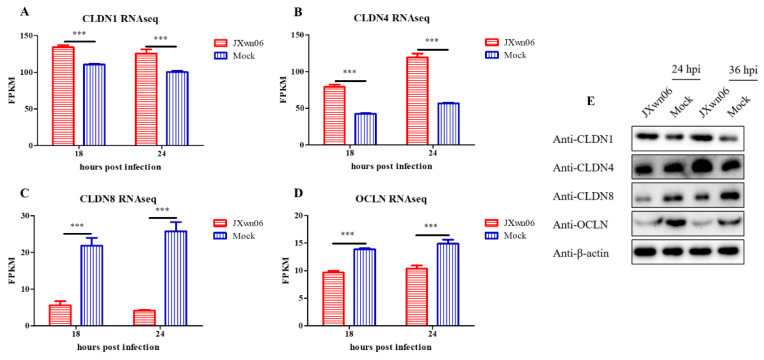
Interendothelial junction-associated genes in PMVECs are dysregulated upon interaction with HP-PRRSV-infected PAMs. RNA-seq data in fragments per kilobase million (FPKM) for four TJ genes, including CLDN1 (**A**), CLDN4 (**B**), CLDN8 (**C**), and OCLN (**D**), for comparison are presented. (**E**) Western blot analysis of CLDN1, CLDN4, CLDN8, and OCLN in co-cultured PMVECs at the indicated time points post inoculation. β-actin served as a loading control. The data are shown as means ± SD (standard deviation), n = 3 independent experiments. Asterisks indicate statistical significance (***, *p* < 0.001).

The RNA-seq data showed that plenty of TJ members were dysregulated in the group with HP-PRRSV-infected PAMs. As shown in Figure 6A–D, compared to mock-infected groups, Claudin 1 (CLDN1) and CLDN4 were significantly upregulated, while CLDN8 and Occludin (OCLN) were downregulated in the HP-PRRSV infection group. The expression levels of these four TJ associated proteins were further determined by Western blotting. As was expected, the expression levels of CLDN1, CLDN4, CLDN8, and OCLN (Figure 6E) at 24 and 36 hpi were conserved with the transcription trend detected by RNA-seq. The expression levels of these TJ proteins were accompanied by the hyperpermeability changes of the PMVECs monolayer in vitro (Figure 5A,B). Taken together, these data suggest that the expression levels of TJ proteins in PMVECs are dysregulated by interactions with HP-PRRSV-infected PAMs, and this process is associated with the destruction of pulmonary microvascular monolayer integrity, which further provides convenience for immune cells flux.

### 3.7. Experimental Validation of Selected Genes

**Figure 7:**
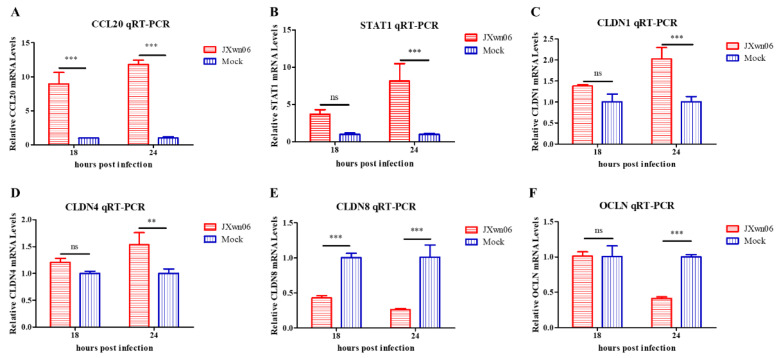
RT-qPCR validation of differentially expressed genes in PMVECs upon interaction with HP-PRRSV-infected PAMs at different time points. Shown are the transcription levels of four upregulated genes (**A**–**D**), and two downregulated ones (**E**,**F**). The levels of these genes were normalized against β-actin and then compared to the mock-infected group. The data are shown as means ± SD (standard deviation), n = 3 independent experiments performed in triplicate. Asterisks indicate statistical significance (ns, *p* > 0.05; **, *p* < 0.01; ***, *p* < 0.001).

To validate the accuracy of transcription level in RNA-seq, the mRNAs of four upregulated genes, including CCL20, STAT1, Claudin 1(CLDN1), and CLDN4, together with two downregulated genes CLDN8 and Occludin (OCLN), were selected to confirm their transcription levels by RT-qPCR with the primers listed in Table 1. As shown in Figure 7A–F, the transcription levels of all four genes were significantly increased compared with the mock-infected group, while those of CLDN8 and OCLN were significantly decreased, with a similar trend with the FPKM value in RNA-seq results.

Therefore, these results demonstrate that, during PRRSV infection, PMVECs function as the pro-inflammatory cells to release abundant pro-inflammatory cytokines and chemokines; at the same time, they are also conducive to the circulating erythrocytes, fluid, and immune cells to flux into the tissues via upregulation of the cell adhesion molecules on the surface of the microvascular wall, as well as disassembling tight junctions.

## 4. Discussion

Clinically, compared with low pathogenic PRRSV (LP-PRRSV) strains, HP-PRRSV infection can cause serious lung lesions, which are primarily characterized by extensive hemorrhage, considerable inflammatory cell infiltration, and pulmonary edema [11], indicating the increased capability of HP-PRRSV to destroy the air–blood barrier of the lungs.

The endothelial cells form a one-cell thick walled layer called the endothelium, which functions as a blood vessel wall and maintains vascular homeostasis. Together with pulmonary epithelium and interstitium, the vascular endothelium constitutes the air–blood barrier that maintains the water–salt balance. However, its functional changes during PRRSV infection are less known. To explore some clues for further study on the roles of PMVECs played in PRRSV-caused lung lesions, the transcriptomic technology was initially carried out, as it is a useful approach to analyze the profile of genome-wide gene expression levels influenced by the investigated factors. It can characterize the transcriptional activity of thousands of genes at once to create a global picture of cell function [35]. In a previous study, endothelial cells have been confirmed to be unsusceptible to PRRSV infection [32]; as a result, the permeability factors secreted by PRRSV-infected PAMs might be the signal for cross-talking with the PMVECs in the endothelial barrier during the viral infection. Thus, a co-culture system with both PMVECs and HP-PRRSV-infected PAMs was set to characterize the dynamic transcriptome landscapes of PMVECs by RNA-seq and comprehensive bioinformatics analysis. Generally, the transcriptome sequencing data indicate that the immune response, metabolic pathways, cell death, cytokine–cytokine receptor interactions, viral responses, and apoptotic processes are significantly regulated upon the interaction with PRRSV-infected PAMs. These significantly regulated genes and enriched pathways are important candidates for further investigation to explore the mechanism of acute lung lesion caused by HP-PRRSV.

Among these pathways, pro-inflammatory cytokines and chemokines, such as IL-1α, CSF3, CCL20, CCL22, and CX3CL2, as well as VCAM-1, are transcriptionally upregulated upon co-culture with HP-PRRSV-infected PAMs, which indicates that the interaction may also lead to excessive inflammatory responses in PMVECs. PRRSV infection has been shown to compromise the integrity of many physiological barriers, including the air–blood barrier, blood–brain barrier, and placental barrier. This process is primarily attributed to exacerbated host immune responses that lead to hyperpermeability of endothelial cells located on the surface of different types of vessels. The increased vascular permeability and pulmonary edema are prominent features of ALI and acute respiratory distress syndrome (ARDS), which are commonly assumed to relate to the levels of critical soluble cytokines, such as vascular endothelial growth factor (VEGF) and TNFα [36,37,38,39]. PAMs secrete a broad range of pro-inflammatory cytokines and chemokines upon PRRSV infection, which are responsible for the severity of pulmonary pathology. Our transcriptomic data further indicate that PMVECs might be important cells in inflammatory responses during PRRSV infection. This is consistent with a recent study that demonstrates that the supernatants of PRRSV-infected primary PAMs can induce significant expression of inflammatory cytokines in vascular endothelial cells [32]. These upregulated molecules in PMVECs further facilitate the hyperpermeability of pulmonary microvascular endothelial cells, influencing disease manifestations.

Besides, several genes of endothelium intercellular junctions were significantly dysregulated in PMVECs, whose products might greatly contribute to maintaining the functions of the endothelial barrier. Degradation of the endothelial junctions has been associated with disease severity in several viral diseases. For example, the expression levels of intercellular junction proteins, including claudin-5, occludin, and zonula occludens-1, are significantly decreased in rabies virus-infected brain microvascular endothelial cells [40]. However, the importance of vascular permeability and the role of intercellular junctions in the pathogenesis of PRRSV are still less reported.

To further explore the mechanism of endothelial leakage, the relationship of vascular permeability and TJ protein expression was also investigated. The TERR and dextran flux assay, two methods reflecting the ability of different particles to cross the endothelium by transcellular or paracellular pathways, were initially used to investigate the endothelial permeability and vascular leakage in vitro. In succession, the transcription and expression levels of TJs in PMVECs were monitored. The results suggested that this interaction between PMVECs and PAMs can exert effects on pulmonary microvascular endothelium, and can then lead to hyperpermeability and endothelial barrier dysfunction. Meanwhile, the dysregulation of TJ proteins, including CLDN1, CLDN4, CLDN8, and OCLN, is confirmed to correlate with the increased permeability of PMVECs in vitro. Given that the presence and integrity of the intercellular junctions are crucial factors for maintaining homeostasis and preserving the contacts between the adjacent endothelial cells of the endothelium to protect them from excessive paracellular movement, TJs might be important molecules to relate with PRRSV-induced pathogenesis. The function and regulation pathway of two important TJs, CLDN4 and CLDN8, during PRRSV infection have been studied in another project of ours.

Due to vascular permeability being affected by multiple factors, except for the disassembly of endothelial junctions, both endothelial cell apoptosis or necrosis and the remodeling of the cytoskeleton might potentially lead to the observed hyperpermeability [41]. However, they are not all analyzed in this study, which might be involved in the future.

**Figure 8:**
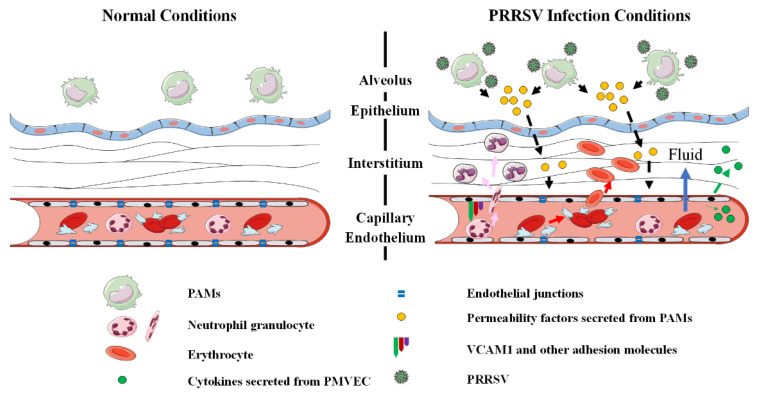
A proposed model for PRRSV dysregulation of the PMVECs barrier. HP-PRRSV-infected PAMs produce secreted permeability factors and use paracrine signaling to communicate with the endothelial barrier during PRRSV infection. PMVECs secrete pro-inflammatory cytokines and express cell adhesion molecules on the surface of pulmonary microvascular endothelium in response to viral infection to aggravate inflammation and monocyte chemotaxis. Conversely, cell–cell junctions between adjacent endothelial cells might be disassembled to promote monocytes, erythrocytes, and fluid flux into tissues.

In conclusion, the data reveal the dynamic transcriptome profiles and functions of DEGs in PMVECs co-cultured with HP-PRRSV-inoculated PAMs in vitro. The results indicate that the secreted permeability factors produced by HP-PRRSV-infected PAMs can disrupt the integrity of the PMVEC barrier by upregulating the expression of monocyte-adhesion molecules and pro-inflammatory cytokines, as well as dysregulation of TJ proteins to facilitate the passage of circulating immune cell and erythrocyte escape vasculature locations to establish hemorrhage and inflammatory cell infiltration (Figure 8). The study provides many valuable clues for further study on the roles of PMVECs in PRRSV-induced ALI, which is one of the key directions to explore the mechanism of PRRSV pathogenesis and reduce PRRSV-induced lesions in the future.

## Figures and Tables

**Table 1:** List of primers used in this study.

Names *	Primer Sequence (5′-3′)
β-actin-F	ACCACCATGTACCCAGGCAT
β-actin-R	GGACTCGTCGTACTCCTGCT
CCL20-F	AAGCAACTTTGACTGCTGCC
CCL20-R	GGATCTGCACACACGGCTAA
STAT1-F	CCATTGGTCCTGAAGACTGGAG
STAT1-R	TTCGTGTGAGTGCCCAAAATG
CLDN1-F	CCGTGCCTTGATGGTAATTG
CLDN1-R	ACCATGCTGTGGCAACTAAG
CLDN4-F	TGGATGATGAGAGCGCCAAG
CLDN4-R	GGGATTGTAGAAGTCGCGGA
CLDN8-F	TGGTGGTGTTGGAATGGTGG
CLDN8-R	GTTGCTTCCAATGAAGGCGG
OCLN-F	GCTGGAGGAAGACTGGAT
OCLN-R	ATCCGCAGATCCCTTAAC

* F represents forward PCR primer; R represents reverse PCR primer.

## Data Availability

The transcriptomic data is available with the link: ftp://ftp.lc-bio.cn/, accessed on 11 January 2022.
